# “We and the nurses are now working with one voice”: How community leaders and health committee members describe their role in Sierra Leone’s Ebola response

**DOI:** 10.1186/s12913-017-2414-x

**Published:** 2017-07-18

**Authors:** Shannon A. McMahon, Lara S. Ho, Kerry Scott, Hannah Brown, Laura Miller, Ruwan Ratnayake, Rashid Ansumana

**Affiliations:** 1 Institute of Public Health, Heidelberg University, Heidelberg, Germany; 2 Associate in International Health, Johns Hopkins School of Public Health, Baltimore, MD USA; 3 The International Rescue Committee, New York, NY USA; 4 Global Health Consultant, Bangalore, India; 5 Anthropology Department, Durham University, Durham, England UK; 6 International Rescue Committee, Freetown, Sierra Leone; 7 Department of Community Health and Clinical Studies, School of Community Health Sciences, Njala University; Mercy Hospital Research Laboratory, Bo, Sierra Leone

**Keywords:** Ebola, Sierra Leone, Village health committee, Qualitative research, Community participation, Health management committee

## Abstract

**Background:**

Across low-income settings, community volunteers and health committee members support the formal health system - both routinely and amid emergencies - by engaging in health services such as referrals and health education. During the 2014–2015 Ebola epidemic, emerging reports suggest that community engagement was instrumental in interrupting transmission. Nevertheless, literature regarding community volunteers’ roles during emergencies generally, and Ebola specifically, is scarce. This research outlines what this cadre of the workforce did, how they coped, and the facilitators and barriers they faced to providing care in Sierra Leone.

**Methods:**

Thirteen focus group discussions (FGD) were conducted with community members (including members of Health Management Committees (HMC)) near the height of the Ebola epidemic in two districts of Sierra Leone: Bo and Kenema. Conducted in either Krio or Mende, each FGD lasted an average of two hours and was led by a trained moderator who was accompanied by a note taker. All FGDs were audio recorded, transcribed, and translated into English by the data collection team. Analysis followed a modified framework approach, which entailed coding (both inductive and deductive), arrangement of codes into themes, and drafting, distribution and discussion of analytic summaries across the study team.

**Results:**

Community volunteers and HMC members described engaging in labor-related tasks (e.g. building isolation structures, digging graves) and administrative/community-outreach tasks (e.g. screening, contact tracing, and encouraging care seeking within facilities). Through their dual orientation as community members and as individuals linked to the health system, respondents described building community trust and support for Ebola prevention and treatment, while also enabling formal health workers to better understand and address people’s fears and needs. Community volunteers’ main concerns included inadequate communication with - and a sense of being forgotten by - the health system, negative perceptions of their role within their communities, and concerns regarding the amount and nature of their compensation.

**Discussion & Conclusion:**

Respondents described commitment to supporting their health system and their communities during the Ebola crisis. The health system could more effectively harness the potential of local responders by recognizing community strengths and weaknesses, as well as community volunteers’ motivations and limitations. Clarifying the roles, responsibilities, and remuneration of health volunteers to the recipients themselves, facility-based staff, and the wider community will enable organizations that partner with health committees to bolster trust, manage expectations, and reinforce collaboration.

## Background

### Overview

Community participation has been promoted as an essential feature of primary health care since the landmark Alma Ata conference in 1978, which stated that “people have the right and duty to participate individually and collectively in the planning and implementation of their health care.” [1]. The literature on community participation in health emphasizes the need for long-term, process-oriented (rather than outcome-oriented) investment in community mobilization [2, 3]. Community participation is also emphasized as a vital feature of emergency response, such as when dealing with epidemics or environmental disasters [4–8]. During disasters, decisions must be made rapidly, resources must be distributed quickly, and the potential for distrust, miscommunication, and conflict are heightened, making inclusive community consultation and capacity building processes especially challenging but nevertheless necessary. This dilemma (of balancing speed with sensitivity to communities affected) was documented by Médecins sans Frontières in their response to the 2005 Marburg hemorrhagic fever outbreak in Angola [9]. The organization ultimately had to forgo burial and disinfection protocols that were technically sound but culturally insensitive in favor of involving local authorities and respected individuals, and accommodating the need for ritual and mourning [9].

The World Health Organization (WHO) described the 2014–2015 Ebola epidemic in western Africa as the “most severe acute public health emergency seen in modern times” [10]. Sierra Leone bore the highest burden of infection, with 8706 confirmed cases and 3956 deaths [11]. Popular media have tended to emphasize community resistance to Ebola management efforts, while the WHO, UNICEF, Action Contre la Faim and the International Rescue Committee (IRC) have highlighted the important role of communities in the Ebola response through, for example, mobilizing peers to carry out preventative measures, encouraging the acceptance of treatment, restricting mobility into and out of communities, and temporarily quarantining suspect cases in the household [8, 12, 13]. Community volunteers and existing community health workers were described as undertaking surveillance activities that enabled the early warning of new transmission chains at the village level [10, 14, 15].

While a cogent description of community-based responses to Ebola was described in urban Liberia, the specific roles of community volunteers and health committees, as well as these actors’ perceptions of their roles during the epidemic has not been extensively documented [13, 16]. The primary objective of this qualitative paper is to describe the roles played by members of Health Management Committees (HMC) and related forms of community-based voluntary engagement during the Ebola outbreak in two districts of Sierra Leone. A secondary objective is to describe the challenges faced by community volunteers in this setting. HMCs and other community representatives were included as participants. For this reason, we highlight that while some perspectives represent insights from individual community members, there is an equal or greater representation of HMC members in our results.

## Methods

### Setting - health volunteers in Sierra Leone

Health management committees (HMCs) exist throughout Sierra Leone, although they are referred to by a variety of names including village development committees, facility management (/monitoring) teams (/committees), and health development committees. These committees are not a standardized intervention across the country and instead take on a range of structures, have different planned and actual roles, and receive support (sometimes monetary and sometimes in-kind) from various non-governmental organizations (NGOs) and government programs. Despite this varied focus, all committees involve a group of volunteers from the community who work together, often in collaboration with health facility staff, to improve community health and give voice to a community’s needs among staff in facilities. In addition to HMC members, there are other health volunteers in many villages who are supported by government programs, development organizations, or religious groups. Furthermore, during the Ebola crisis, contract tracers were selected from the community and paid a small allowance; oftentimes these individuals had a pre-existing relationship to the health system (working as porters, for example). As has been documented in several African settings, such volunteers often occupy an ambiguous position between formal and informal labor, for example they are paid stipends that do not constitute a wage but may nonetheless be very valuable in local terms [17]. Volunteer positions are often coveted forms of status [17].

### Data collection

Table 1 presents a timeline of events related to data collection and analysis. A team of eight data collectors with previous experience in qualitative research on behalf of NGOs or community development sectors were trained for three days. Following training and piloting, data collection teams conducted focus group discussions (FGD) at eight peripheral health units in urban and rural settings across Bo and Kenema districts, which represented areas of low and high Ebola transmission, respectively. The research team asked the head of each health facility (called the "in-charge") to invite their HMC members to participate in an FGD on the day of data collection. In some cases, if not enough HMC members were available, other community members involved in the health facility were asked to participate. HMC typically include a community chief or headman, a mammy queen (i.e., a female leader), a teacher, and several health mobilizers. If, upon arriving to a community, the research team noted that women had been excluded from FGD participation, the team requested that participants collectively identify and invite women to join. While our data includes an oversampling of male perspectives, at least one woman was present in each FGD. Two FGDs were conducted at each facility, one in mid-December 2014 and one in late January 2015. Prior to the start of FGDs, data collectors introduced themselves and described the purpose of the study; they then sought (and in all cases received) informed consent from each respondent. Following consent, participants were invited to outline a set of ground rules to be employed during the FGD. The most common ground rules included that all opinions be respected, all information shared would remain private, each participant should feel free to speak, and no person should be interrupted. Drinks and snacks were then distributed and the FGD formally began. Questions employed during FGDs included: "Please tell us about your work in relation to the health of this community" and "How has your work changed in light of Ebola?" and "How do you feel about the work done by HMCs in this community?" Throughout data collection, research managers conducted nightly debriefings wherein the team reviewed their field notes, shared highlights from the FGDs, identified emerging themes, debated further lines of inquiry, and troubleshot challenges. FGDs lasted an average of two hours, and while it is difficult to determine whether rapport was built, in several instances respondents thanked the research team for asking the community to share their thoughts about life amid Ebola. FGDs were audiorecorded and transcribed verbatim in Krio or Mende before being translated into English.

**Table 1:** Timeline of events related to data collection and analysis

Nov 2014	Train data collectors
Nov 2014	Conduct pilot testing
Dec 2014	Data collection round 1 • Focus groups discussions
Jan 2015	Data collection round 2 • Focus groups discussions
Jan-Feb 2015	Transcription and translation
Mar 2015	Data analysis: coding • Conduct inductive and deductive coding based in part on a priori codes (developed from the questionnaire) and open-coding (developed as relevant data emerged from transcripts)
Mar 2015	Data analysis: matrix development • Collapsing and arranging codes (along with their attendant quotes) across transcripts
Apr 2015	Data analysis: writing analytic summaries • Synthesis of text across domains of the matrix
Apr 2015	Data analysis: distribution, feedback and discussion of analytic summaries
Apr 2015	Literature review
May 2015	Consensus regarding content of results section

### Data analysis

Three FGDs were lost during the transcription and translation process and could not be included in analysis. Beginning in March 2015, the first author coded 13 FGDs following an adapted framework approach, a “matrix-based analytic method” that facilitates the sorting and synthesis of data around themes as well as comparisons across districts [18]. The FGDs covered multiple topics including impressions of and experiences with Ebola, experiences with infection prevention, and the roles of respondents and committees in managing Ebola. This line of questioning informed the development of broad themes during the analysis process. Using Microsoft Excel, the study team generated a matrix with a row for each FGD and a column for each of our 20 broad themes. Each cell in the matrix contained coded quotations from the FGD transcripts relevant to the column’s theme.

After developing this framework, the lead author generated outputs of all quotations collected under each theme, re-read these quotations, and wrote analytic summaries that detailed basic themes emerging from the data. For example, quotations under the initial broad theme “buckets for hand-washing” resulted in the creation of several basic themes such as “encourage community to hand-wash,” “construct hand-washing stations,” “explain to community how to create chlorine water solution for hand-washing,” and “ensure that those entering the clinic wash their hands first”. This detailed analytic summary was shared with the broader research team and reviewed in detail by the third author, who simultaneously conducted a literature review. Lead authors then collaboratively grouped the basic themes into organizing themes (such as “physical labor during Ebola,” “health-oriented tasks,” and “representing the health system to the community”), and five global themes, which serve as the sub-headings in our results section.

### Ethical approval

The overall study from which the data are sourced received ethical approval from Institutional Review Boards of Durham University and the Sierra Leone Ethics and Scientific Review Committee.

## Results

We divided findings into five global themes, which outline the roles of individuals and HMCs before and during Ebola, and the barriers and facilitators encountered by respondents during the emergency response (Table 2).

**Table 2:** Summary of key findings for each global theme

Global theme	Key findings
1. Pre-Ebola community context and respondent activities	• HMCs were active: regular meetings, some fundraising, promotion of health-related behaviors, and engagement with health workers
2. Respondent activities during Ebola	• Manual labour (e.g. building walls for the clinic, cleaning facilities, digging graves, manning checkpoints) • Administration or outreach (e.g. maintaining records, contact tracing, conducting screenings) • Some tasks involved navigating tense interactions with other community members
3. Respondent role providing social mediation between health system and community during Ebola	• Explained community concerns and fears to health care workers (e.g. personal protective equipment (PPE) and burial) • Asked health workers delicate or embarrassing questions on behalf of the community (e.g. whether Ebola could be sexually transmitted) • Explained the value of practices promoted by health workers to the community (e.g. the need for screening and isolation) • Sought to build community trust in the health system
4. Respondent sources of motivation and facilitators of action during Ebola	• Intrinsic sources of motivation included a desire to serve and lead, fear of Ebola, and pride/trust in one’s health facility and health providers • Extrinsic sources included compensation, recognition of the government’s limited capacity, recognition of Ebola’s severity, and NGO support
5. Respondent sources of discouragement and barriers to action during Ebola	• Intrinsic sources of discouragement included sadness, grief, and loneliness, fear of contracting Ebola, concern that the government had forgotten them • Extrinsic sources included community misconceptions about their payment and community anger at them for “collaborating” with the health system

### Pre-Ebola community context and respondent activities

Respondents reported a high level of activity in the months and years before the Ebola outbreak. They described holding regular meetings to discuss “matters pertaining to the health facility” and to communicate with the broader community about health issues. One participant in Kenema mentioned: “Every month we call an HMC meeting with other community members to inform them about all that is happening within the realm of health activities in this chiefdom” (− FGD, Kenema District, December 2014). Some were actively fundraising to improve health facilities by, for example, constructing a maternal waiting home. Other HMCs passed bylaws to encourage health-related behaviors, for instance leveraging a fine on lactating women who missed postnatal clinic dates.

Community outreach and education were central to respondent activities even before Ebola. Respondents encouraged community members to use facilities and recognized that "the health facility … belongs to the community" (− FGD, Bo District, December 2014). Respondents also described traveling with health workers during outreach activities, or recording health and census related information about catchment areas. Respondents described collaborative relationships with facility staff. When new drugs and supplies arrived at the clinics, health facility staff counted stock with respondents “for transparency’s sake.”

Respondents supported health workers by seeking to solve problems faced by providers, and informing providers of health issues in the community:

We interact with the health workers to know their problems, and in some circumstances we intervene to resolve differences. We also find information relating to health care conditions of the villagers and promptly inform the health workers for their intervention. – FGD, Kenema District, December 2014

### Respondent activities during Ebola

During the Ebola epidemic, respondents described a range of public health promotion tasks, which can be grouped into those involving manual labor and those involving to more so administrative duties or community outreach. Some activities were coordinated and compensated through programs supported by NGOs or the government, while others sprang from respondents’ own initiative.

Labor-related tasks included manually walling off clinic compounds, setting up screening booths at compound entrances, cleaning health facilities and public spaces (sometimes spraying chlorine), building hand-washing stations and isolation areas, digging graves to bury deceased community members, and digging pits in which to burn contaminated materials (such as blankets from homes or medical equipment from health facilities). Community members provided labor to run checkpoints on roads around their villages in order to enforce travel bans. Manning checkpoints not only involved a large time commitment but also the unpleasant tasks of demanding identification documents from travelers, trying to prevent people from “slipping around” the checkpoint, and at times turning away people coming from regions with Ebola. In a minority of FGDs, respondents described bearing a financial cost for provisions necessary to keep checkpoints running.

Tasks more directly linked to administrative duties and community outreach included maintaining health records, conducting contact tracing, undertaking home visits to find ill community members, notifying burial teams of deaths, and conducting screenings outside health facilities. Record keeping involved working closely with nurses to record details of non-Ebola deaths during the Ebola outbreak, such as what treatment the deceased had received and what illnesses they may have had, and reporting it to the authorities. In the role of contact tracing, tracers identified those who had come into contact with someone infected with Ebola, monitored the appearance of symptoms and conveyed the information to health authorities.

Screening was a multi-step process, and was discussed in the most detail by all respondents. Screening and was one of several activities that placed respondents at the frontline of interaction with potential Ebola patients. The process involved spraying the screening area with a chlorine solution, asking anyone seeking to enter the clinic premises to wash their hands in chlorinated water, taking a visitor’s temperature with a digital thermometer, and informing those with high temperatures to wait in case they were warm from exertion. If a second reading indicated a high temperature, or if an individual showed additional signs or symptoms of Ebola, respondents described being trained to send the visitor to an isolation area and notify health facility staff.

Similar to running the checkpoints, running screening booths at times placed respondents in tense interactions with community members. While respondents explained that visitors to facilities were eventually willing to participate in hand washing and screening processes, these requests initially induced suspicion and resistance: “The previous times when they came and you would tell them to go through those procedures they would say we are practicing *juju* (witchcraft). So they don’t come. They take their children home. But it is better now.” (− FGD, Bo District, January 2015).

### Social mediation across health providers, burial teams and community members

Respondents described engaging in many tasks to facilitate communication between the community and providers. More explicitly, respondents described conveying community concerns about Ebola transmission, personal protective equipment (PPE), community-held fears of infrared thermometers (“laser guns”), and the role of burial teams. Respondents also described how they felt well positioned to ask delicate or embarrassing questions, which the community was not comfortable asking in a health encounter; for example, one respondent described how he could ask whether Ebola could be sexually transmitted. In terms of representing the health system to the community, respondents described how they could relate to the community while conveying frightening information. For example, like many community members, respondents described initial skepticism regarding the veracity of Ebola, and they questioned the necessity of screening and isolation. They explained that they came to understand the reality and severity of the disease through first hand exposure to friends and family falling sick and dying. In addition, their access to biomedical information (through clinic meetings and trainings) regarding how Ebola spreads and how it can be prevented built their confidence in the medical system’s response. Having personally undergone this change in understanding, respondents described feeling uniquely positioned to empathize with community concerns while simultaneously encouraging care seeking in facilities, explaining medical responses to Ebola, addressing community fears of health workers and fostering compassion toward health workers on behalf of the community.

Respondents described in depth how they conveyed community concerns to providers (and vice versa) regarding two issues specifically: PPE and burial processes. PPE (plastic equipment that covers the wearer’s face and body) was worn by both health workers and members of burial teams. Respondents urged providers to recognize that seeing a medical provider dressed similarly to an individual tasked with burying dead bodies was profoundly disturbing. Respondents also highlighted in conversations with providers that the experience of watching a body being taken away without having been prayed upon undermined efforts to encourage families to come forward when a family member died. In conversations with communities, respondents described how PPE was essential to stopping the spread of Ebola. Respondents’ understanding of the value of burial teams enabled them to encourage community members to accept new burial practices, but to also mediate between “burial boys” and community members in tense encounters. Respondents described intervening between distressed community members and exhausted burial teams to prevent altercations.

(Burial teams) had no respect in dealing with the dead. When somebody dies, they will just put the person in the body bag and throw the corpse in a hole. Even when carrying the corpse to a gravesite, they will just drop the body any which way they feel when they are tired. At one time, that nearly created a fight here if it was not our quick intervention. – FGD, Bo District, January 2015

Respondents described how community bylaws were passed to support the Ebola response, often in response to requests for support from health professionals, specifically to (1) prohibit families from harboring sick members, and (2) discourage stigmatization (in the event of Ebola survivors returning home). Community members who violated these bylaws could be fined. Respondents spread news of the bylaws through door-to-door visits, took action to enforce the bylaws, and sought to encourage their peers to seek medical help and to welcome Ebola survivors back to the community. On this latter point, respondents described arranging or partaking in ceremonies to publicly welcome survivors back to the community (“We welcome them by dancing with them” – FGD, Bo District, December 2014).

Canvassing communities to identify those who are sick and encouraging clinic visits required extensive efforts to overcome community fear of visiting health centers, which communities generally viewed as Ebola transmission points. One respondent recalled a time when he followed up with a sick friend who attempted to run away, and was able to convince the friend to visit the health center for testing:

My friend … was trying to escape from this town when he fell and collapsed and he was brought here. As soon as he gained consciousness he rose and ran into the bushes. We had to find him and I especially had to encourage him … After doing his test he was not Ebola positive but rather had pneumonia. Even up to now any time he sees me he will thank me for giving him courage to go through the process. – FGD, Bo District, December 2014

### Sources of motivation and facilitators to HMC action during Ebola

Respondents described several intrinsic sources of motivation. Individuals spoke of a desire to serve and lead, dedication to community, fear of Ebola, and recognition that self-sufficiency was necessary. The most salient motivating feature across FGDs was a sense of pride for one’s health facility and trust or loyalty toward an admired health provider. Respondents described several occasions when their health center in-charge took action to protect the community from Ebola. One respondent said “God bless our in-charge” when recalling how the in-charge had arranged to have a burial team’s PPE properly disposed of after the burial team left it in the middle of the road. Finally, respondents described how pre-existing relationships with providers enabled collaboration during Ebola and compelled them to try harder to listen to and trust health providers’ Ebola messaging.

Another thing again, initially we had no knowledge about Ebola. We were told not to touch dead people or even treat our sick relatives. We thought they were telling lies. But this man our health provider came to our level and explained to us the effect of this disease. He also shared with us every detail he gets from meetings that he attended. - FGD, Bo District, December 2014

So we and the nurses are now working with one voice. So there is nothing happening with regards to this disease that they [the nurses] don’t inform us [the tracers] about because they are at the center. When there is an emergency they will call us first and inform us before they send the report. - FGD, Bo District, January 2015

Extrinsic motivation included financial or in-kind compensation for meeting or workshop attendance, recognition of the government’s limited human resource capacity to manage crises, recognition of the severity of Ebola, and NGO supervision, direction, and support. Respondents mentioned that frequent outside inspections of their Ebola-prevention activities also encouraged them to work hard. Along with appreciating monetary compensation for involvement, respondents also described enjoying the support and direction gleaned by comparing experiences with other participants at “NGO workshops”.

### Sources of discouragement and barriers to HMC action during Ebola

Respondents endured discouraging interactions and encountered barriers to their Ebola-prevention efforts. Intrinsic discouragement stemmed from the sadness, grief, and loneliness many were confronting in relation to the loss of loved ones, personal fear of contracting Ebola, concern that the health system had forgotten them, and frustration with the way Ebola imposed a standstill on life in general (forcing the immediate stop of other construction and sensitization activities, for example). Extrinsic barriers included inadequate supplies and resources, criticism and distrust from their community, and concerns or misunderstandings about the purpose of a task. Contact tracers in particular highlighted that they were to receive weekly allowances but that this payment was irregular.

Community misconceptions included suspicion that respondents received payment for their work (which was true for community tracers but not the rest of HMC members) and community anger toward respondents for “collaborating” with the health system.

They accused us of collaborating with the health workers that Ebola is here. See, we are “liars.” That is what we were called. – FGD, Kenema District, December 2014

Some factors, such as fear that the country’s health system was ill-equipped to manage the Ebola crisis, proved to be simultaneously motivating and discouraging. A crippled health system motivated respondents because they could see an immediate and profound need for their services, yet it also discouraged them because they felt frustrated, overwhelmed, and distraught about the fate of their country.

## Discussion

This paper contributes to a modest but growing body of literature on the role of community members [8, 13, 16, 19] and formalized volunteer cadres [20] in responding to emergency situations generally, and the Ebola epidemic specifically. In overstretched, under-resourced health systems, it is vital to understand both *what* these entities have been able to do, and *why/how* they have been able to do these things (enabling features/mechanisms) including contextual factors that support or challenge functionality [21, 22]. Such understanding may enable the quick, appropriate and systematic engagement of health volunteers and health committees during public health emergencies, rather than ad-hoc and incomplete approaches.

Our research during the epidemic documented significant community mobilization, some of which was driven by pre-existing health committee members who assumed responsibilities to protect their communities and to support health system responses to Ebola. Respondents described extensive inputs of physical labor and served a range of social roles. They communicated Ebola-related messages to their peers, enhanced provider understandings of community fears, and advocated for community needs within the health system. Enabling mechanisms that supported community action included the dual orientation of health committee members as community-members and health system-affiliates. This dual role built community support for Ebola prevention and treatment activities and enabled health workers to better understand and respond to community concerns and fears. This finding demonstrates the necessity of communication during emergencies wherein the roles, responsibilities, and remuneration of committee members is made clearer among not only health committee members but also providers and the community generally.

Three broader contextual factors may have contributed to the high self-reported level of HMC functionality during the Ebola outbreak. First, the pre-existing relationships between the HMCs and health workers, which developed in non-emergency periods, bolstered health committee willingness to work with the health system. HMC members framed their trust in health workers within broader narratives of efforts to improve access to healthcare since the end of the civil war. Second, external inputs, in this case workshops provided by IRC and others and the arrival of infection prevention and control supplies, focused HMC efforts and gave members clear direction and purpose. Third, the unique horror of Ebola, and the recognition that outside intervention would not be enough to protect the community, galvanized community action.

Although the role of health committees during the Ebola epidemic has not been systematically examined in the academic literature, Oxfam has reported in the grey literature on their work in Sierra Leone setting up committees during the Ebola outbreak [23]. The organization worked in four districts (Western Area, Kailahun, Freetown and Koinadugu) alongside District Health Management Teams and District Ebola Response Coordination to set up 821 health committees [23]. Similar to our findings, the Oxfam-initiated committees identified barriers to prevention, case management, and safe burial. They also developed action plans to overcome these barriers. Oxfam reported that actively involving health committees in the Ebola response was essential to their work and that people wanted to see community health committee activities continue after Ebola.

Findings from this study stress that public health systems should plan to ensure that pre-existing community committees and groups have specific roles during emergencies. Community engagement during emergency preparedness and response has been emphasized in high-income settings, including with indigenous communities in remote areas of Australia [24] and Canada [25]. Schoch-Spana et al. [26] report that “structured dialogue, joint problem-solving, and collaborative action among formal authorities, citizens at-large, and local opinion leaders” during a crisis such as influenza has a range of beneficial outcomes including improving officials’ ability to govern, the application of communally-held resources, and the mitigation of community-wide losses. However, researchers have noted that health department capacity for community engagement requires dedicated time and financial support to build long-term, trusting relationships [27]. In low-income countries such as Sierra Leone, sustained support of health committees is currently provided by NGOs such as Oxfam and IRC.

Since the conclusion of the Ebola epidemic, the government of Sierra Leone has highlighted the importance of drawing on communities to promote health in several documents, most prominently in the “Basic Package of Essential Health Services 2015-2020” [28]. The package highlights a need for more actors who can serve as interlocutors between communities and facilities, particularly in the aftermath of Ebola when facility-community tensions were heightened. Focusing on Community Health Workers (CHWs) as key partners to fulfill this role, the package states that the government is codifying CHW training, allocating resources for a national scale-up of CHW programming, and it is in the process of formally including CHWs within the Ministry of Health’s workforce. While these are promising steps, the Package gives comparatively little attention to the role of other community-level actors such as HMCs. The package recognizes that such groups exist, but the document falls short of outlining how these groups could be more coordinated, formally recognized, and compensated for their efforts whether amid health emergencies or in routine health care promotion [29].

Health committees have the potential to improve health system quality, coverage, and accountability in non-emergency periods [21, 30] and were a central component of the Bamako Initiative [28, 31]. However, harnessing this potential, and extending it to emergency preparedness, requires ongoing health system support to ensure that community involvement includes marginalized groups, that health workers have the incentives and resources to work with committees, and that committees have genuine control over some decisions and resources. We summarize the key “lessons learned” that arose from our research to inform health systems strengthening and public health emergency response in Table 3.

**Table 3:** Lessons learned for health systems strengthening and emergency response

1.	Community leaders, volunteers, and health committee members can perform vital functions during public health emergencies
2.	The importance of community leaders, volunteers, and health committee members rests not only in their capacity to carry out manual labor and administrative tasks, but also in their capacity to mediate between communities and the health system
3.	Positive pre-existing relationships between communities and health workers are a key enabler for community volunteers to engage in difficult tasks during crises, particularly tasks that violate social norms (e.g. burial rituals)
4.	During emergencies, the resilience and capacity of community leaders, volunteers, and health committee members can be supported by ensuring clarity among stakeholders about compensation, reassuring community workers that they are not forgotten, providing trainings and equipment, and creating spaces for dialogue between health workers and community workers

### Limitations and opportunities for further research

This paper presents self-reported roles and experiences of community members, many of whom were part of HMCs. We lack data to triangulate these reports, and respondents may have emphasized or exaggerated the effectiveness of their activities to provide socially desirable responses. HMCs in Kenema (but not Bo) were established in the 2000s as part of an IRC-supported reproductive healthcare program. Although there were no outward signs of data collectors’ IRC affiliation, respondents in Kenema may have sensed or assumed that data collectors were affiliated with IRC and may have then been motivated to present themselves in a positive light. However, the HMCs in Bo were not supported by IRC, and self-reported activities of Bo HMC members did not substantively differ from Kenema members. Overall, we feel the responses represent the range of activities undertaken by community members, but not the effectiveness or frequency of these activities.

We do not have data on how other community members, health care providers, or burial teams felt about respondents’ stated activities, which is meaningful given the amount of partnering necessary as an interlocutor between the groups (enforcing bylaws, contact tracing, managing screening and checkpoints, mediating conflicts, and going door-to-door to stop families from keeping sick members at home). Respondents reported that community members’ initial reservations regarding infection prevention measures eventually waned, but this may not align with community perspectives. Furthermore, while respondents described camaraderie with facility staff, we do not know if this was reciprocated. Future research could explore how communities and providers view health committees and could work to devise effective, responsible oversight mechanisms.

Our respondents were mostly members of HMCs, as identified by health facility staff, other community at large, or other members of the HMC. It is often not possible to tell within the transcripts if an individual is responding as a representative of an HMC, another entity, or as an individual. While there is often a clear distinction among NGOs between work that is conducted as an HMC member versus work conducted as hired assistants, respondents were less discerning regarding whether their activities were done in an official HMC capacity or in another capacity. For example, while the IRC paid community health workers to contact burial teams in the event of a death and to conduct screening (and did not consider these tasks as inherent to the HMC experience), respondents who were paid saw the tasks as intertwined with their community health worker role.

We could not assess inclusivity of HMCs (i.e. how members came to join the HMC, which community members were excluded) and how the membership mix of the HMC may have influenced their activity. While it is possible that the Ebola emergency compelled communities to temporarily disregard internal power inequalities and struggles, it is also possible that respondents chose not to discuss these issues during the FGDs for a range of reasons (such as an inability to speak freely in a group setting, to avoid exacerbating tensions, or to present a positive image of their group in hopes of future NGO engagement), or because they were not explicitly asked about it.

Finally, we want to underscore that this data was collected at or around the height of the Ebola epidemic; the ability to maintain or sustain high levels of motivation as an epidemic wanes or ends merits further research.

## Conclusion

Despite the above study limitations, it is valuable to document the self-reported experiences and perspectives of community members involved in the grassroots Ebola response. This paper highlights community resilience and the high value placed on self-sufficiency in a time of fear, danger, and loss within a broader context of health system strain and a legacy of civil war. Health system responses to public health emergencies must engage community actors, particularly health committee members, not only for their physical labor and their ability to support health providers but also for their capacity as social mediators. The development and support of health committees can be an important aspect of health system strengthening, which enables resilience and responsiveness during crisis periods. Health committees play a number of valuable roles in non-emergency periods and, as illustrated in this paper, in emergencies health committees provide vital community linkages and resources.
